# Preference for Cannibalism and Ontogenetic Constraints in Competitive Ability of Piscivorous Top Predators

**DOI:** 10.1371/journal.pone.0070404

**Published:** 2013-07-19

**Authors:** Pär Byström, Per Ask, Jens Andersson, Lennart Persson

**Affiliations:** Department of Ecology and Environmental Science, Umeå University, Umeå, Sweden; University of Toronto, Canada

## Abstract

Occurrence of cannibalism and inferior competitive ability of predators compared to their prey have been suggested to promote coexistence in size-structured intraguild predation (IGP) systems. The intrinsic size-structure of fish provides the necessary prerequisites to test whether the above mechanisms are general features of species interactions in fish communities where IGP is common. We first experimentally tested whether Arctic char (*Salvelinus alpinus*) were more efficient as a cannibal than as an interspecific predator on the prey fish ninespine stickleback (*Pungitius pungitius*) and whether ninespine stickleback were a more efficient competitor on the shared zooplankton prey than its predator, Arctic char. Secondly, we performed a literature survey to evaluate if piscivores in general are more efficient as cannibals than as interspecific predators and whether piscivores are inferior competitors on shared resources compared to their prey fish species. Both controlled pool experiments and outdoor pond experiments showed that char imposed a higher mortality on YOY char than on ninespine sticklebacks, suggesting that piscivorous char is a more efficient cannibal than interspecific predator. Estimates of size dependent attack rates on zooplankton further showed a consistently higher attack rate of ninespine sticklebacks compared to similar sized char on zooplankton, suggesting that ninespine stickleback is a more efficient competitor than char on zooplankton resources. The literature survey showed that piscivorous top consumers generally selected conspecifics over interspecific prey, and that prey species are competitively superior compared to juvenile piscivorous species in the zooplankton niche. We suggest that the observed selectivity for cannibal prey over interspecific prey and the competitive advantage of prey species over juvenile piscivores are common features in fish communities and that the observed selectivity for cannibalism over interspecific prey has the potential to mediate coexistence in size structured intraguild predation systems.

## Introduction

The large intraspecific size variation in aquatic communities determines to a large extent the trophic relationships between species and increases the likelihood of cannibalism, omnivory and intra guild predation (IGP) [1]–[6]. A general preference for cannibalism over interspecific predation may have major implications for coexistence between predators and prey as density - dependent cannibalism may reduce predation on other species within the community [3]. This self-regulating mechanism of cannibalism has, for example, been theoretically shown to increase the stability of predator-prey systems [7]. Furthermore, in systems where predators and prey compete for shared resources, cannibalism in the top predator increases possible environmental conditions for coexistence [4], [8]. In addition, coexistence in IGP systems has been suggested to be strongly dependent on the intermediate consumer being a superior resource competitor [9]–[11]. Still, recent theoretical studies have shown that coexistence in stage-structured IGP systems may occur even when the IG predator is the superior resource competitor and coexistence is in this case facilitated by stage structure or by cannibalism in the IG predator [6].

Both predatory and competitive interactions are crucially dependent on body size [12], [13]. As individuals grow in size, interactions change in strength and competitive interactions among juveniles may also shift to predator-prey interactions over ontogeny [1], [14]. It has been suggested that species that undergo substantial niche shifts over their ontogeny will be less efficient in each niche than species that specialize in a specific niche due to ontogenetic covariance [14], [15]. For example, in fish, piscivores may be inferior competitors on zooplankton compared to planktivore specialists because juvenile piscivores are burdened with morphology and behaviour more adapted for piscivory than for planktivory [1], [16]. Although this pattern of covariance in species performance over ontogeny has long been suggested, there are still surprisingly few documented examples [1], [16]. Covariance within the piscivore niche may also be possible as variation in piscivore efficiency between interspecific and intraspecific (i.e. cannibalism) predation may involve similar constraints. Foraging efficiency of piscivores may be hypothesized to be higher on conspecifics than on other prey fish species because of covariance in both morphology and behaviour [17]. In one of the few studies addressing this issue, Juanes [18] accordingly showed that conspecific prey body size was larger than other prey fish species in the diets of piscivores, which was suggested to reflect a higher capture success on conspecific prey. Hence, both ontogenetic constraints in competitive ability and a general preference for cannibalism in piscivorous top predators can be suggested to be major mechanisms promoting patterns of coexistence in fish communities.

In this study, two approaches were used to address the above two hypotheses. First, we experimentally tested whether Arctic char *Salvelinus alpinus* (L.) is a more efficient cannibal than interspecific predator, using ninespine sticklebacks *Pungitius pungitius* (L.) as interspecific prey and whether ninespine stickleback is a better resource competitors than juvenile char. Second, a literature survey was carried out to test to what extent there is a general pattern in the suggested covariances in competitive ability and piscivory efficiency on conspecifics versus other prey fish species. The survey focused on a) the preference for cannibalism by adult piscivores on conspecific juveniles relative to interspecific predation on similar sized prey species and b) the competitive ability measured as foraging efficiency on zooplankton between small piscivores and their prey fish. Finally, the implications of these relationships are discussed in relation to coexistence in general and in fish communities with piscivorous top consumers, in particular.

## Materials and Methods

### Species studied

Arctic char has a circumpolar distribution and is the dominant fish species in many subarctic lakes in the northern hemisphere [19]. In many cases char is the only fish species within these lakes, but may also be found to coexist with e.g. ninespine sticklebacks [19]–[20]. Char is omnivorous, feeding on zooplankton and benthic invertebrates as well as on small prey fish including conspecifics and cannibalism has also been suggested to be a major structuring force in char populations [21]–[24]. Ninespine stickleback feed on a wide range of resources from zooplankton to small fish fry and eggs [25] and has been shown to have strong effects on zooplankton communities [19], [26], [27]. Ninespine sticklebacks have also been suggested to be an important prey fish for many piscivorous species including Arctic char [20], [28].

### Experimental studies

Ninespine sticklebacks used in all experiments were collected with traps from Lake Hamptjärn (63°52′34″N, 20°12′51″E). Ninespine stickleback is the only fish species present in that lake. The small char used as intraspecific prey were all raised in a commercial rearing station and were offspring's of wild parents. Hence, both ninespine sticklebacks and small char used as prey in the experiments had no prior experience of piscivorous predators. Both small char and ninespine sticklebacks were kept in large holding tanks prior to introductions into experimental treatments and fed a mixture of live zooplankton and frozen chironomids. The piscivorous char used in all experiments were offspring of wild parents and brought up in a commercial rearing station. To allow them to acclimatize to natural resources and conditions and to develop into functional piscivores, the char used in the pond experiments were kept over winter in a neighbouring pond with both natural invertebrate resources and stocked ninespine stickleback and small char at equal densities of approximately 0.6 individuals per m^2^ which are within the range of YOY char densities found in the near shore habitats in natural systems [29]. Char used for selectivity experiments in the pool experiments (n = 4) were held in pairs in two circular holding tanks (900 l) and fed with live stickleback and YOY char two weeks prior to experiments to ensure that they were functional piscivores and had the capacity to feed on both prey types. All experiments were conducted in late May early June at water temperatures between 11–16°C (outdoor pond experiments) and 16.5–17°C (indoor pool experiment). May early June is also a period in Umeå, Sweden (63°49′N, 20°18′E) when in principal, there is 24 hours of daylight conditions.

Permission to collect sticklebacks with traps in Lake Hamptjärn for the experimental studies was given via telephone by the land owner. Sampling methods, collection of experimental fish, method of sacrifices and design of all experiments in this study comply with the current laws of Sweden and were approved by the local ethics committee of the Swedish National Board for Laboratory Animals in Umeå. (CFN, license no. A-19-06 to corresponding author, Pär Byström).

#### Pool experiment

One indoor wading pool (Ø 3.0 m, height 0.5 m, water depth 0.42 m) was used to test selectivity of Arctic char between intraspecific prey and ninespine sticklebacks. The colour of the inside wall and bottom was black and light was provided above from a fluorescent lamp with a continuous 24 h light cycle. No refuges or shelter for prey fish were present in the pool as we explicitly wanted to test piscivorous capacity of adult char without any confounding effects of behavioral differences in the prey types. Two pairs of piscivorous char were used (pair 1: 277±6 mm, 178±10 g; pair 2: 283±2 mm, 188±4 g, mean ±1SD). The pairs of piscivorous char were starved for 32 hours prior to introduction into the pool at 08.30–09.30 in the morning. In the afternoon, half an hour before introduction of prey fish the piscivorous char pair was gently forced into a holding chamber and thereafter the desired density and identity of prey fish were introduced to the pool. Between 16.00–16.30 the piscivorous char pair was then released and allowed to feed for 20 h. The morning after at 08.00–08.30 the pool were sampled for surviving prey fish and the piscivorous pair was captured and replaced with the second pair of piscivorous Arctic char. Predator pairs were only used once for each prey type combination and surviving prey was not used in subsequent trials. Three different prey fish treatments was used; 40 YOY char alone (replicated 2 times), 40 sticklebacks alone (n = 2) and a sympatric treatment of 20 YOY char and 20 sticklebacks (n = 2).

#### Pond experiment and piscivorous attack rate estimates

Five experiments were conducted to investigate the piscivory efficiency of char when feeding on small char and ninespine stickleback (Table 1). Experiments were either conducted in a pond (80×25 m, mean depth 0.7), with enclosures of a size of 12×6 m (experiment 1 and 2), for details see [30] or in two similar ponds (32×10.8 m, mean depth 0.9 m each) divided into eight enclosures (4×10.8 m) (experiments 3–5), for details see [29]. The environment in the ponds provided access for prey fish of natural refuges in terms of structural complexity of the heterogeneous soft bottom and a narrow vegetation belt of *Carex* sp. at the shore line. In all experiment two piscovorous char were used part, from experiment 1, when only one piscovorous char were used (Table 1). In all experiments piscivorous char were introduced 10 to 20 days prior to introduction of prey. Corresponding control treatments without piscivorous char for estimating background mortality was conducted in all experiments. At the end of each experiment all remaining fish were collected with a seine net and enclosures were considered to be empty when three subsequent empty pulls of the seine net were obtained.

**Table 1:** Summary of conducted predation experiments of Arctic char feeding on char and sticklebacks, showing; number of replicates for treatments with predators and controls for background mortality, number of piscivorous char used per enclosure, numbers and density of prey species introduced, mean predator and prey sizes ±1 SD, duration of experiments, time (month) when experiments were conducted, average temperature based on mean of start and end temperatures of experiments.

Experi-ment	Enclosure volume (m^3^)	# replicates (Exp., Control)	# predators/enclosure	Predator size (mm)	Prey species	# and density (# m^−2^) prey	Prey size (mm)	Duration (days)	Average temp (°C) Month
1	50.4	3.1	1	289±37	char	60, 0.8	46.0±4.1	7	12 May-June
2	50.4	5, 2	2	284±38	char	32, 0.4	82.0±6.2	7	12 May
3	38.9	6, 2	2	248±18	char	100, 2.3	44.7±3.7	14	n.a. June
4	38,9	6, 2	2	292±10	char	150, 3.5	28.8±2.3	7	14.7
		6, 2	2		char + stick.	100, 2.3+50, 1.2	28.8±2.3, 45.9±2.4		June
5	38.9	3, 2	2	289±11	stick.	70, 1.9	39.2±2.5	14	16.0
		3, 2			stick.	70, 1.9	52.9±3.0		June

Average temperature is not available for experiment 3.

To standardize piscivorous char efficiency on conspecific and interspecific prey we calculated char attack rate using a Hollings type I functional response curve assuming that predators were not saturated and that prey mortality is a function of both predation and background mortality. Victim mortality was then expressed as: 
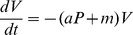
(1)where *V* is victim density, *P* predator density, *a* the attack rate of single predator, *t* is the duration of the experiment and *m* is the background mortality of the prey. Rearranging equation 1 gives the expression for predator attack rate, *a*, in equation 2. 
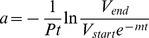
(2)where *V_start_* and *V_end_* represents the density of victims at the start and at the end of the experiment, respectively and background mortality, *m*, is given by equation 3. 
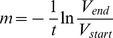
(3)


#### Foraging experiments and zooplankton attack rate estimates

In order to test whether ninespine stickleback is a more efficient resource competitor than small char, the attack rates on zooplankton were estimated for ninespine stickleback and contrasted with previously published attack rates estimates of char feeding on *Daphnia sp.* of similar size and origin used in this experiment [31]. Four size classes of sticklebacks were used (25.4±1.7, 33.5±1.4, 44.8±2.3 and 59.6±2.0 mm, mean ±1 SD) and fed frozen chironomids and live zooplankton captured from a pond nearby Umeå university. After a minimum of one week, six individuals of each size class were placed in 30 L aquariums with a water temperature of 14°C. The back and the sides of the aquaria were covered with dark grey plastic and 11 W fluorescent tubes was placed 50 cm above the water surface of the aquaria. During the acclimation period of 2 days, sticklebacks were fed once a day with live zooplankton (mainly *Daphnia*). Thereafter, the sticklebacks were trained for another two days in the experimental procedure.

The experimental procedure was as follows. The sticklebacks were starved for 24 h prior to each experimental trial. Immediately before a trial, the individual was gently forced into a small holding chamber at one side of the aquaria and thereafter the desired density of *Daphnia* (size: 1.11±0.08 mm, mean ±SD) was gently poured from above into the aquaria. When zooplankton were evenly distributed, the stickleback was released from the holding chamber and measurements of capture rates started when the stickleback captured the first prey and the time was measured until the capture of second to eighth prey (dependent on size of ninespine stickleback and density of prey) had been consumed. To estimate the attack rates, the capture rate estimates at different prey densities were fitted to a Hollings type II functional response using the same procedure as described for char in [31].

### Literature survey

Web of Science® was used to search for studies that either directly presented selectivity indices for cannibalism and interspecific predation or studies that presented data that allowed for calculation of Ivlev's index of selectivity [32]. Ivlev's index ranges between 1 and −1, where positive values indicates preference, negative values avoidance and values close to 0 indicate that prey is eaten in proportion to environmental densities. The first search was designed to include records containing the key word “piscivory” in combination with either or both of the key words “selectivity” and “preference”. In a second, more detailed search the key words: “cannibalism”, “intraspecific predation”, “selectivity” and “preference” were combined together with the common names of known cannibalistic piscivores. This search targeted species that were commonly cited in the litterature: Smallmouth bass (*Micropterus dolomieu* Lacépède); Largemouth bass (*Micropterus salmoides* Lacepéde); Arctic char; Brown trout (*Salmo trutta* L.); Eurasian perch (*Perca fluviatilis* L); Yellow perch (*Perca flavescens* Mitchill); Pikeperch (*Sander lucioperca* L.); Cod (*Gadus morhua* L.); Hake (*Merluccius merluccius* L.); Cape hake (*Merluccius capensis* Castelnau) and Walleye Pollock (*Theragra chalcogramma* Pallas). Cannibalism was found in all of the above species. Most frequently, retrieved records reported only diet data with no reference to environmental densities of prey and consequently did, in most cases, not allow for estimates of the preference for cannibal prey relative to interspecific prey. For the piscivorous fish species where information on cannibalism and interspecific predation could be obtained, a literature search was conducted to find studies reporting on competitive relationships between these piscivorous species and their potential competing prey species. In the search the common name of the piscivorous fish species were combined with either of or in combination with the key words: “interspecific competition”, “resource competition” and the common name of the respective competitive prey species. These searches returned few studies where resource competition between piscivorous fish species and their competitive prey species had been addressed. The studies that were found mostly reported on dietary and niche overlap between species (the potential for competition between species) and seldom presented data for comparison of the competitive ability on shared resources between the species.

### Statistics

Differences in consumption of different prey species in the pool experiment were analysed with T-test statistics and number of consumed prey were Ln (x+1) transformed prior to analysis to standardise variance between treatments. Differences in char attack rate on prey types were analysed with standard ANCOVA's using prey type as factor and prey length as covariate to control for size effects between prey types. Similarly, to test for differences in attack rate on zooplankton between char and sticklebacks, we used standard ANCOVA's with char/stickleback as factor and length of consumer as covariate to control for size effects between consumers. Non parametric directional one-tailed Sign test were used to test the hypothesis that piscivores are more efficient on conspecific prey than on interspecific prey and that piscivores are less efficient than their prey species when feeding on zooplankton.

## Results

### Experimental studies

#### Pool experiment

**Figure 1:**
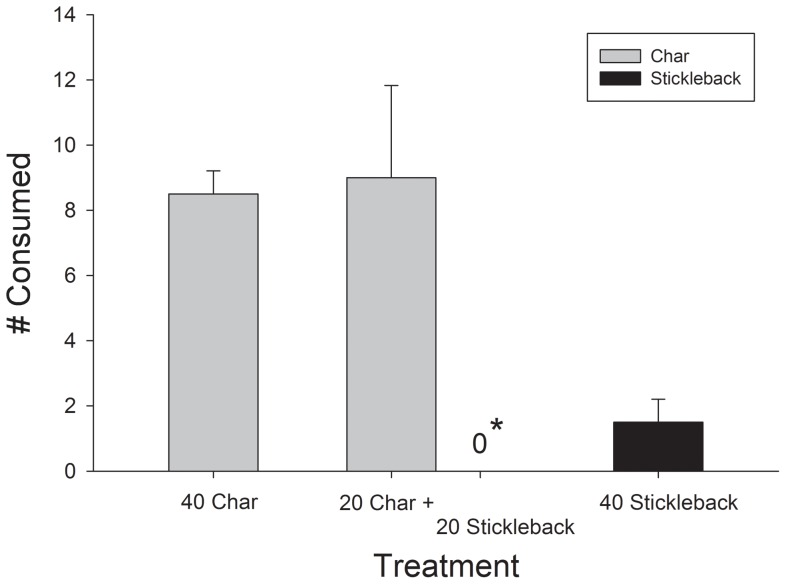
Number of consumed prey (small char and/or nine spine stickleback) in the different treatments in the pool experiment. * No (0) sticklebacks were consumed in the sympatric treatment.

Piscivorous char consumed only conspecific prey (small char) and no interspecific prey (sticklebacks) when offered equal densities of both prey types (paired T-test, *T* = 42.7, df = 1, *P* = 0.015) (Fig. 1). When offered either prey type, piscivorous char consumed sticklebacks although at very low number and number of consumed prey was higher for conspecific than for interspecific prey (T-test, *T* = 4.84, df = 2, *P* = 0.04) (Fig. 1).

#### Pond experiment

**Figure 2:**
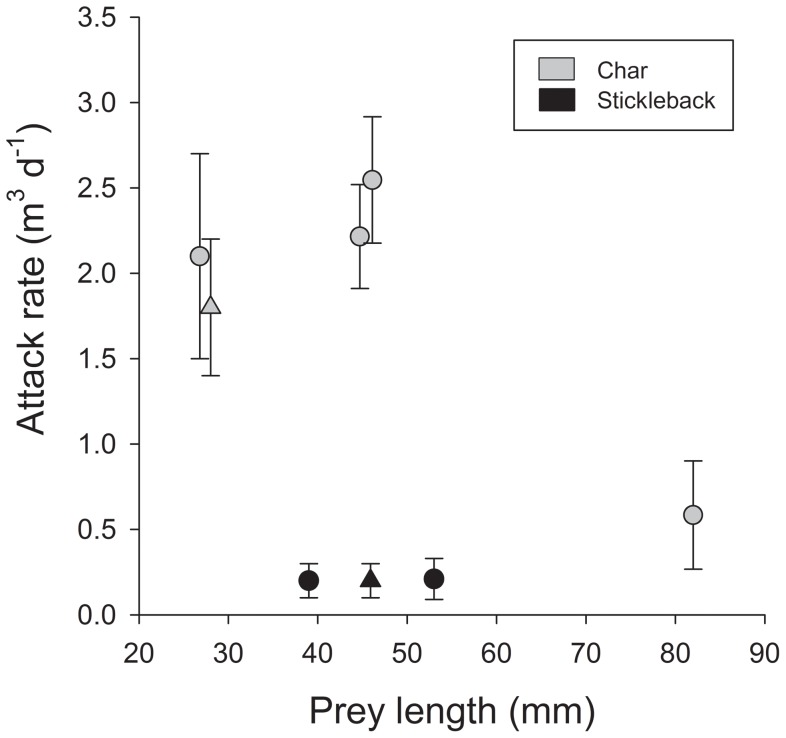
Attack rate (m^3^ day^−1^±1SD) of piscivorous char when feeding on small char and/or ninespine sticklebacks. Circles denote singles prey species treatments and triangles mix prey species treatment.

When foraging either on conspecifics or interspecific prey, char were more efficient on conspecific than on interspecific prey, as observed in the consistently higher attack rate on different sizes of char than on ninespine stickleback (ANCOVA, using body size as covariate, main effect *F*_1,3_ = 16.3, *P* = 0.027) (Fig. 2). Similarly, when both prey types were present in the same enclosures estimated attack rates on char were higher than that on sticklebacks (paired T-test, *T* = 10.8, df = 5, *P*<0.001) and attack rates were similar for both prey categories as in single species experiments (Fig. 2).

**Figure 3:**
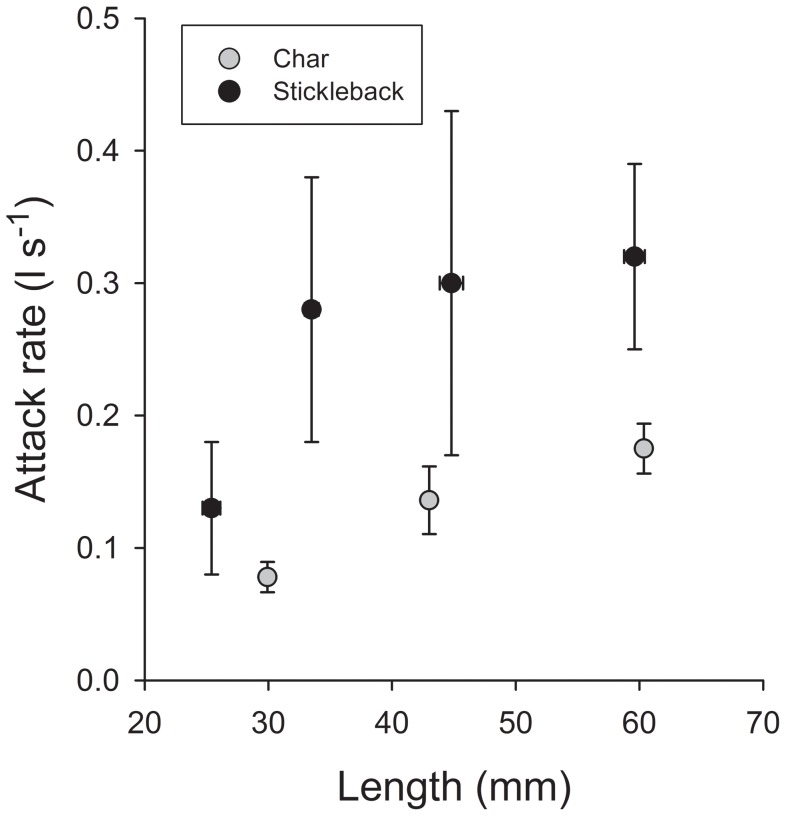
Attack rate (l s^−1^±1SE) of char (obtained from [31]) and ninespine stickleback when feeding on zooplankton (*Daphnia sp.*).

Comparison of zooplankton attack rate estimates of ninespine stickleback with previous estimates for small char, showed that ninespine stickleback was a more efficient zooplanktivore than char (ANCOVA, using body size as covariate, main effect *F*_1,4_ = 16.3, *P* = 0.016) (Fig. 3).

### Literature survey

Eight studies (covering in total ten top predators-prey species interactions) were found that either presented data that allowed to calculate selectivity between cannibal and interspecific prey (five studies) or that directly reported the selectivity between cannibal and interspecific prey in piscivorous fish species (three studies) (Table 2). The data cover five different families of piscivorous fishes (Centrarchidae, Gadidae, Merluccidae, Percidae, Salmonidae). There was a clear preference for cannibalistic prey compared to interspecific prey (8 out of 11 cases, including the experimental study in this paper) (one-tailed, Sign test: *P* = 0.012). Exceptions were predation by closely related piscivores (Percidae) on YOY perch and YOY pike-perch where there was no clear preference for either prey type [36], [37]. One study showed clear selection for interspecific prey, where Largemouth bass displayed stronger selectivity for cichlids than for small conspecifics. Still, in the same study largemouth bass also displayed a preference for cannibalism compared to interspecific predation on five different cyprinid species (Table 2).

**Table 2:** Preference between cannibalism (Ca) and interspecific predation (Ipr) in eight species of piscivorous fish.

Species pair	Ivlev's index of Selectivity		Source
	Ca	Ipr	
*M. capensis* - **	+	−	[33]
*M. Merluccius* - *	+	−	[34]
*M. salmoides* - Cyprinidae	0.51	−0.71	[35]
*M. salmoides* – Cichlidae	−0.20	0.11	[35]
*P. fluviatilis* – *R. rutilus*	0.30	−0.72	[17]
*P. fluviatilis* - *S. luciperca*	0.00	0.03	[36]
*S. alpinus* - *P. pungitius* ^†^	+	−	Present study
*S. luciperca* - *P. fluviatilis*	0.12	0.06	[37]
*S. luciperca* – *R. rutilus*	0.43	−0.85	[37]
*S. trutta*- *S. alpinus*	0.58	−0.13	[38]
*T. chalcogramma* – *C. pallasi*	0.36	−0.98	[39]

*preference for cannibalism over six different interspecific prey types.

**Interspecific prey was not specified.

*, ** confer source for information on selectivity index. + and - indicates positive and negative selectivity.

Data for comparing competitive ability between piscivores and their prey species could be obtained for the five species (including the experimental study in this paper) in the comparison of cannibalism and interspecific predation (Table 3). Piscivore species were competitively inferior compared to their competing prey species for the zooplankton resource in all cases (one-tailed, Sign test: *P* = 0.031) (Table 3).

**Table 3:** Comparison of competitive ability (attack rate) on shared zooplankton prey between piscivores and prey species.

Species pair	Competitive ability Piscivore (pi) vs. Prey (p)	Source
*P. fluviatilis* - *R. rutilus*	pi<p	[40], [41]
*M. salmoides* – *L. macrochirus*	pi<p	[42]
*S. trutta* – *S. alpinus*	pi<p	[43]
*S. luciperca* – *A. brama*	pi<p	[44], [45]
*S. alpinus* – *P. pungitius*	pi<p	Present study, [31]

## Discussion

### Intra- versus interspecific predation

The predation experiments showed that piscivorous Arctic char was a more efficient cannibal than interspecific predator on ninespine stickleback. This was evident both in controlled short term pool experiment and at a longer time scale in semi natural environments with natural more complex bottom substrate and resource availability for prey. Our experimental results and derived estimate of predation mortality also corresponded to a clear selection for cannibalistic prey over interspecific prey that we found in the literature survey. Although available data regarding the relationship between cannibalism and interspecific predation is limited, the fact that the pattern is consistent over several different families of piscivorous fish species, across both marine and freshwater habitats suggests that the apparent selectivity for cannibalism may be a general feature of piscivorous fish species and in any case provides important implications for coexistence in fish communities in certain systems. Prey species often demonstrate well developed anti predator defences in both morphology and behaviour [46]. Juanes [18] suggested that the trend for consuming larger conspecifics compared to interspecific prey amongst piscivores indicates that there is a higher capture rate of cannibal prey relative to alternative interspecific prey types, which is supported by the experimental study in this paper. There was only one case in the literature survey where interspecific prey was clearly selected over cannibal prey. In contrast to the other studies which involved species naturally occurring together and may be considered as natural coevolved systems of piscivores and their prey, this was a case of an introduced population of largemouth bass where largemouth bass had higher selectivity for cichlids over conspecific prey [35]. Moreover, largemouth bass in the same study also selected for cannibal prey over cyprinids. It is possible that the selectivity between cannibalism and interspecific predation in this study occurred because the cichlid prey species were not coevolved with the introduced largemouth bass and that cichlids in general appears to be very sensitive to introduced predators (e.g. extinction of cichlids in lake Victoria due to introduction of Nile perch (*Lates niloticus* L.) in [47]).

The literature survey presented some evidence for that piscivore preference was stronger for closely related prey species compared to more distantly related species. For example, the related percidae species pikeperch and perch only showed small differences in preference for each others juveniles compared to their own juveniles, whereas both species showed strong preference for cannibalism compared to interspecific predation on roach (*Rutilus rutilus* L.) (Table 2). Furthermore, largemouth bass showed a stronger preference for prey species from the more closely related family Cichlidae compared to Cyprinidae, whereas brown trout still showed strong preference for cannibal prey over juveniles of the closely related Arctic char (Table 2). These results suggest that increasing phylogenetic distance between species, and thereby increasing differences in behaviour and morphology, may affect the relative strength of interspecific predation and cannibalism.

Cannibalism may favour species competing for shared resources with juvenile cannibals by decreasing the density of juvenile piscivorous competitors [48], [49]. Correspondingly, a higher efficiency of cannibalism compared to interspecific predation has been suggested to theoretically increase conditional range for coexistence in communities with cannibalistic predators [4], [5]. Although parameter space for coexistence is also predicted to increase in presence of cannibalism over a wide range of predator mortality rates on consumers [8], we anticipate that piscivore efficiency is affected by habitat structure which may shift the relative vulnerability of prey species in different habitats mediated by behavioural and morphological anti-predator adaptations in specific prey species [17], [50]. Furthermore, increasing habitat complexity may also induce species specific changes in the foraging tactics of piscivores that potentially changes the relative vulnerability of different prey species [51]. Consequently, the impact of cannibalism on species coexistence may vary dependent on the composition of the fish assemblage and the structure and distribution of the physical habitat in the system.

### Predation - competition trade-offs and coexistence in IGP systems

The foraging experiments presented in this study showed that ninespine sticklebacks are more efficient than juvenile char when foraging on zooplankton. The higher competitive ability of intermediate species compared to piscivorous species that is found our experiment and in our literature survey corresponds to the predictions based on ontogenetic covariance [1], [14]. The negative impact of competitive prey species on the juvenile stages of piscivorous fish species has been proposed since long and has important implications for competition in size-structured populations with competing specialists and generalist predators [1], [16]. Still, the literature survey provided only four studies that addressed the competitive asymmetry between competitive prey species and piscivorous fish species. Olson et al. [42] provide an compelling example and show that bluegill (*Lepomus Macrochiru* Rafinesque) is a stronger competitor than largemouth bass using both experimental data and data from natural populations. Similarly, perch [40], [41] and brown trout [43] have lower foraging efficiency on zooplankton resources compared to their competing prey species. This competitive disadvantage of piscivores relative to their competing prey may induce juvenile competitive bottlenecks in the piscivore, which may reduce growth of juvenile piscivores and decrease recruitment into the adult piscivorous stage [41], [43], [52].

In systems where omnivorous top predators compete with intermediate species for shared resources, i.e. IGP systems [53], coexistence has been suggested to be dependent on the competitive superiority of the intermediate species and also to be limited to intermediate levels of productivity [9]–[11]. Recent theoretical work has further shown that coexistence in IGP systems may be dependent on the presence of cannibalism in the top consumer and the parameter range with coexistence between omnivorous top predators and intermediate species in IGP systems is substantially increased when the IG predator is cannibalistic [4], [8]. In conclusion, selectivity for cannibalism over interspecific prey in top consumers and inferiority in exploitation of zooplankton resources compared to their competing prey, that appears to be the case based on the results from this study, can be advanced to be mechanisms promoting the coexistence of IGP systems in fish communities.
